# Workplace Determinants of Social Capital: Cross-Sectional and Longitudinal Evidence from a Finnish Cohort Study

**DOI:** 10.1371/journal.pone.0065846

**Published:** 2013-06-11

**Authors:** Tuula Oksanen, Ichiro Kawachi, Anne Kouvonen, Soshi Takao, Etsuji Suzuki, Marianna Virtanen, Jaana Pentti, Mika Kivimäki, Jussi Vahtera

**Affiliations:** 1 Finnish Institute of Occupational Health, Turku and Helsinki, Finland; 2 Department of Social and Behavioral Sciences, Harvard School of Public Health, Boston, Massachusetts, United States of America; 3 School of Sociology, Social Policy and Social Work, Queen’s University Belfast, Belfast, United Kingdom; 4 UKCRC Centre of Excellence for Public Health (NI), Queen’s University Belfast, Belfast, United Kingdom; 5 Department of Epidemiology, Graduate School of Medicine, Dentistry and Pharmaceutical Sciences, Okayama University, Okayama, Japan; 6 Department of Epidemiology and Public Health, University College London, London, United Kingdom; 7 Institute of Behavioral Sciences, University of Helsinki, Helsinki, Finland; 8 Department of Public Health, University of Turku and Turku University Hospital, Turku, Finland; The University of Hong Kong, Hong Kong

## Abstract

**Objective:**

To examine which contextual features of the workplace are associated with social capital.

**Methods:**

This is a cohort study of 43,167 employees in 3090 Finnish public sector workplaces who responded to a survey of individual workplace social capital in 2000–02 (response rate 68%). We used ecometrics approach to estimate social capital of work units. Features of the workplace were work unit's demographic and employment patterns and size, obtained from employers' administrative records. We used multilevel-multinomial logistic regression models to examine cross-sectionally whether these features were associated with social capital between individuals and work units. Fixed effects models were used for longitudinal analyses in a subsample of 12,108 individuals to examine the effects of changes in workplace characteristics on changes in social capital between 2000 and 2004.

**Results:**

After adjustment for individual characteristics, an increase in work unit size reduced the odds of high levels of individual workplace social capital (odds ratio 0.94, 95% confidence interval 0.91–0.98 per 30-person-year increase). A 20% increase in the proportion of manual and male employees reduced the odds of high levels of social capital by 8% and 23%, respectively. A 30% increase in temporary employees and a 20% increase in employee turnover were associated with 11% (95% confidence interval 1.04–1.17) and 24% (95% confidence interval 1.18–1.30) higher odds of having high levels of social capital respectively). Results from fixed effects models within individuals, adjusted for time-varying covariates, and from social capital of the work units yielded consistent results.

**Conclusions:**

These findings suggest that workplace social capital is contextually patterned. Workplace demographic and employment patterns as well as the size of the work unit are important in understanding variations in workplace social capital between individuals and workplaces.

## Introduction

Social capital refers to features of social organisation such as networks, norms, and social trust that facilitate coordination and cooperation for mutual benefit [1]. During the last two decades, researchers have focused on social capital as one of the social determinants of population health [2], [3]. Building social capital has been suggested as an avenue to promote health and to reduce health disparities within and between populations [4]. However, suggestions on how to intervene in order to build social capital have been sparse, partly owing to the paucity of research on factors that determine the levels of social capital.

Theoretically, social capital literature comprises at least two separate overlapping paths: the individual level (or micro-level) approach of social capital emphasising individual perceptions of the level of cohesion or solidarity of the group to which the individual belongs to and the macro-level approach defining social capital as a collective resource of a neighbourhood or a workplace [5]. Accordingly, social capital has been measured both at the individual level and at the group level.

Various researchers have suggested that at the individual level, social capital is determined by socio-economic factors, such as education, socioeconomic status (SES), employment status[6]–[10], and behavioural tendencies such as altruism [11]. This proposition may be problematic given that social capital is also likely to be determined by the social context in which the individual is embedded [12]. Focusing on the individual as the "level of analysis" ignores the contextual and relational nature of social capital. Indeed, some multilevel analyses have suggested that community variations in levels of social capital cannot solely be attributed to the compositional characteristics of individuals [8], [13]. Conceptually, social capital reflects the structure of social relationships and it may be considered a feature of the collectives to which the individuals belong [12]. These include families, neighbourhoods, communities, societies, and workplaces. Thus, there is a need to examine the characteristics of the social environment (in the language of multilevel analysis, what we call "level 2 and beyond") as drivers of social capital.

Recently, several studies have specifically begun to study the contextual determinants of social capital, including urban sprawl [14], [15], neighbourhood walkability [16]–[19], and loss of off-sale alcohol outlets [20]. Workplace as a source of social capital is a promising line of research although the findings so far have been confined to neighbourhoods and communities and not included workplace which might nowadays be particularly important source of social capital [6]. In earlier studies, low workplace social capital has been associated with sub-optimal health, depression, hypertension, and mortality[21]–[24]. In the present study, we aimed at examining this issue by showing the determinants of social capital in the workplaces. We hypothesised that also in the workplace, the characteristics of the environment (context) are associated with the level of social capital. Limited theory on contextual features exist that may be relevant to accumulation of social capital. Nonetheless, the theory proposes that social capital inheres in social relations facilitating social interaction [6], [34], [45]. Therefore, we hypothesised that in the workplace context, the factors that enhance social interaction are associated with the accumulation of workplace social capital.

Therefore, the aim of our study was to investigate which contextual factors are important in the development of social capital in the workplace in the Finnish Public Sector Study which afforded us with data on contextual workplace characteristics, such as demographic and employment patterns and work unit size of more than 3000 workplaces and 40,000 employees in these workplaces.

## Methods

### Participants and Design

Data on employees and workplaces were obtained from the Finnish Public Sector Study involving personnel in 10 municipalities and 21 hospitals in Finland. The study was approved by the Ethics Committee of the Finnish Institute of Occupational Health. Baseline survey questionnaires addressing workplace social capital and other factors were mailed to employees in 2000–2002, and 48,598 responded (response rate 68%). Of them, 48,003 were found in the employers’ administrative records.

We linked these participants to their work units (a total of 3576 work units at the lowest level of organisational hierarchy, such as a kindergarten or a hospital ward) based on employers' administrative records. These records provided us with characteristics of each workplace. We excluded participants with missing information on workplace social capital, work unit characteristics, or covariates (n = 4161) and participants working in units with less than 3 respondents (n = 675) because social capital can be accrued only via group membership. The number of participants with missing values in each covariate and social capital measure varied between 0 and 1425 per variable; this is 0% to 3% of the participants (missing value in any of the variables among 8.7% of the participants). Thus, there was not any particular variable with excess number of missing values. Thus, the main analytical sample consisted of 43,167 employees nested in 3090 work units (range 3−382 employees per work unit). The mean response rate across the work units was 71% (interquartile range 57%−87%). In only 14% of the work units, less than half of the employees responded to the survey.

Of all baseline respondents, 36,440 were still employed in the target organisations and of them 29,180 responded to a follow-up survey in 2004 (response rate 80%). Of them, workplace social capital changed for 12,108 individuals between the two time points. These participants with repeated responses to workplace social capital comprised a subsample for the longitudinal investigation of the effect of change in characteristics of the work unit on change in workplace social capital within individuals.

### Measures

#### Individual workplace social capital

We assessed workplace social capital with an 8-item measure, specifically designed to assess social capital in the workplace. The measure has been shown to be a reliable and valid indicator of workplace social capital [25], and is associated with self-rated health, depression, hypertension, and mortality [21]–[24]. Using a 5-point Likert-scale, participants reported their perceptions of the following issues: "We have a 'we are together' attitude", "People feel understood and accepted by each other", "People keep each other informed about work-related issues in the work unit", "Members of the work unit build on each other's ideas in order to achieve the best possible outcome", "People in the work unit cooperate in order to help develop and apply new ideas", "We can trust our supervisor", "Our supervisor treats us with kindness and consideration", and "Our supervisor shows concern for our rights as an employee". The internal consistency of the scale was acceptable (Cronbach's alpha 0.88). We used the mean of the eight ratings.

#### Aggregated work unit social capital

We aggregated social capital to the work unit level by applying an ecometrics approach (see statistical analyses section below for details) [26]. Thus, in addition to individual social capital at work, we calculated work unit means adjusted for individual variables. The work unit social capital measure showed good ecometric properties: the intra work unit correlelation coefficient (ICC) was 0.24 and the reliability coefficient of the workplace social capital scale score was 0.71. We divided both scores into three groups using interquartile range: low (<Q1), moderate (Q1–Q3), and high (>Q3) levels.

#### Work unit characteristics

As previously [21], we calculated the annual mean of the following work unit characteristics at baseline: size (the number of person-years done in the work unit); the proportion of male employees, fixed-term employees, and manual employees (the proportion of person-years done by these groups in the work unit); the percentage of sick days from working days; and employee turnover (1 minus the ratio of person-years to employee count). All these work unit-level characteristics were treated as continuous variables in the analyses. The choice of the work unit characteristics was partly on theoretical grounds on their importance in explaining variation of the effect of social capital on health [21], and partly by availability.

#### Covariates

We obtained sex, age, occupational position, and type of job contract (permanent vs. fixed-term) from employers' records. We used three indicators of SES: occupational position, education, and residence size. Occupational position was assessed using the occupational-title classification of Statistics Finland: high (upper-grade non-manual workers, e.g. physicians, teachers), intermediate (lower-grade non-manual workers, e.g. technicians, registered nurses), and low (manual workers, e.g. cleaners, maintenance workers). Data on highest educational attainment was derived from Statistics Finland and categorised according to International Standard Classification of Education (ISCED) 1997 to primary (class 1), secondary (classes 2–4), and tertiary education (classes 5–8). Population Registration Centre provided us with data on size of residence divided into small (<70 m^2^), medium (70–100 m^2^), and large (>100 m^2^) [27]. Marital status (married or cohabiting vs. other), health and psychological distress were obtained from survey responses. Self-rated health (very good or good vs. less) and psychological distress measured by a 12-item version of the General Health Questionnaire (scores ≥4 vs. less) [28], [29] were included in the analysis because poor physical or mental health could hamper social connections and participation in the workplace and affect ratings of the level of social capital.

### Statistical Analyses

#### Ecometrics approach

We used an ecometrics approach to calculate a new outcome variable at the work unit level [26]. The measurement of workplace characteristics based on individual responses is inherently multilevel; to account for this we followed ecometric methods. Thus, instead of simply aggregating the survey data to work unit level and computing a workplace score for the social capital measure, a 3-level regression model was applied to derive the workplace measure of social capital [30]. In the ecometrics apporach, individual social capital items (1^st^ level) were nested within individuals (2^nd^ level) who were nested within work units (3^rd^ level). Using this 3-level model, we applied mixed models to calculate the work unit level residuals of the social capital measurement adjusting for all individual level covariates, that is, sex, age, type of job contract, occupation, education, residence size, marital status, self-rated health and psychological distress.The residuals comprised the differences of adjusted work unit level means from the total mean. The residuals, which represented the part of workplace social capital that cannot be attributed to individual response patterns, consituted the work unit level social capital variable that was used in the analyses of social capital in the 3090 work units [26], [31]. From the 3-level model, we also calculated the ecometric properties: the intra class correlation (ICC) coefficient for work units and work unit reliability coefficient.

#### The main analyses

We used two complementary methods to model the association of a change in each work unit characteristic and individual workplace social capital: multilevel and fixed effects multinomial logistic regression for unordered categorical data. We also investigated the associations of the work unit characteristics and the ecometric-based work unit-level social capital in 3090 work units using multinomial logistic regression models. In all models, we calculated the unit of change to represent approximately the size of interquartile range in the respective variable (Table 1).

**Table 1:** Sample characteristics at baseline in 2000–02; the Finnish Public Sector study.

Individual characteristics	N (%)
Age (mean, SD) years	44.6 (9.4)
Sex	
Men	8166 (18.9)
Women	35,001 (81.1)
Occupational position	
High (Upper grade non-manual)	12,493 (28.9)
Intermediate (Lower grade non- manual)	22,734 (52.7)
Low (Manual)	7940 (18.4)
Highest educational attainment	
Tertiary education	23,614 (54.7)
Secondary education	14,835 (34.4)
Primary education	4718 (10.9)
Residence size	
Large (>100 m^2^)	14,793 (34.3)
Medium (70–100 m^2^)	16,266 (37.7)
Small (<70 m^2^)	12,108 (28.0)
Marital status	
Married or cohabiting	32,808 (76.0)
Other	10,359 (24.0)
Type of job contract	
Permanent	35,561 (82.4)
Fixed-term	7606 (17.6)
Self-rated health	
Good	31,735 (73.5)
Poor	11,432 (26.5)
Psychological distress	
No	32,088 (74.3)
Yes	11,079 (25.7)
**Work unit characteristics**	Median (IQ range)
Size (person-years)	28.4 (18.0–51.6)
Rate of sick leaves (%)	4.1 (2.7–5.8)
Proportion of male employees (%)	10.0 (2.0–28.0)
Proportion of temporary employees (%)	25.0 (16.0–34.0)
Proportion of manual employees (%)	4.0 (0–22.0)
Employee turnover (%)	35.2 (22.4–46.8)

SD; standard deviation, IQ; interquartile.

#### Multilevel analyses

Multilevel modelling approach is appropriate for data that involves units at a lower level (43,167 employees) nested within units at a higher level (3090 work units) [32]. We conducted 2-level hierarchical models to study the contextual effects of work unit characteristics on individual social capital at work.

First, we conducted multilevel multinomial logistic regression models using generalised logit model to examine the associations of a predicted change in each work unit characteristic and individual workplace social capital controlling for all individual covariates in these models. This approach allowed for examination whether work unit factors had an impact on social capital between individuals over and above the effects of individual level variables. We set the lowest levels of workplace social capital as the referent group and estimated a multinomial regression model for a pre-determined increase in each work unit characteristic for high and moderate in relation to low levels of workplace social capital.

#### Fixed effects analyses

We included responses from repeat data in the fixed effects analyses which were complementary to the multilevel multinomial analyses of cross-sectional data. Fixed effects models are regression models to examine within-individual changes. In within-subject analyses, the aim is to examine whether the change in work unit characteristics (exposure) and the change in social capital (outcome) are to the same direction. They can be used to analyze longitudinal data with repeated measurements of both independent and dependent variables if the predictor variable of interest changes in value across two time points for a substantial portion of the sample population [33]. In the subsample (n = 12,108) whose workplace social capital changed, for the majority of them a work unit characteristic also changed (55% to 99% by characteristic) between the two time points. Hence, these 12,108 individuals were involved in the fixed effects analyses. Because each individual is used as his/her own control, the fixed effects models control for all time invariant characteristics of the individuals, such as sex and occupational status, we only adjusted for time-varying covariates of age, self-rated health, psychological distress, and size of residence.

#### Work unit level analyses

Finally, we conducted multinomial logistic regression models at the work unit level using the ecometrics-based social capital variable in 3090 work units. This approach allowed for the examination whether any of the work unit characteristics was also associated with social capital of the work units.

The results are presented as odds ratios (ORs) and their 95% confidence intervals (CIs). The statistical analyses were calculated with SAS© 9.2 statistical software (SAS Institute, Inc, Cary, NC, USA) using proc GLIMMIX, proc SURVEYLOGISTIC, and proc MIXED procedures.

## Results

At baseline, the study sample comprised of 8166 male and 35,001 female employees, whose mean age was 44.6 years (range 18–64 years) (Table 1). Eighteen percent were manual workers and 18% had a fixed-term job contract. Three quarters of the participants reported good health and lack of psychological distress. Half of the participants were working in work units with fewer than 29 employees, where less than 4% of the employees were manual, 10% male and 25% employees were on a fixed-term contract. In half of the work units, the percentage of sick leaves was more than 4.1% and the annual turnover more than 35.2%.

Table 2 shows the results of associations between work unit characteristics and individual social capital from multilevel multinomial logistic regression models. Every 30-person-year increase in the size of a work unit reduced the likelihood of the workplace belonging to the highest (compared to the lowest) category of workplace social capital by 6% (OR 0.94, 95% CI 0.91–0.98) when all individual level covariates were controlled for. A 20% increase in manual or male employees was associated 8%–23% lower likelihood of high levels of workplace social capital, whereas a similar increase in fixed-term employees increased the likelihood of high levels of workplace social capital by 11%. Net of covariates, a 20% increase in employee turnover increased the probability of high levels of workplace social capital by one fourth (OR 1.24, 95% CI 1.18–1.30) and moderate levels by 9% (OR 1.09, 95% CI 1.05–1.13). These associations attenuated but remained significant when all work unit characteristics were simultaneously entered in the model (ORs 1.18, 95% CI 1.11–1.25 and OR 1.07, 95% CI 1.03–1.11, respectively). Percentage of employees having a sick leave episode was not associated with social capital in the workplace.

**Table 2:** Associations between work unit characteristics and individual-level social capital at workplace: adjusted odds ratios and their 95% confidence intervals from multilevel multinomial regression models* (n = 43,167) and fixed effects multinomial regression models** (n = 12,108); the Finnish Public Sector study.

	Moderate vs. low levels of workplace social capital	High vs. low levels of workplace social capital
**Work unit characteristic**		
Work unit size/30 person-years' increase		
Between individuals*	0.99 (0.97–1.01)	0.94 (0.91–0.98)
Within individuals**	0.99 (0.98–1.00)	0.98 (0.97–0.99)
Rate of sick leaves/3% increase		
Between individuals*	0.96 (0.93–1.00)	0.96 (0.91–1.01)
Within individuals**	0.97 (0.94–0.99)	0.96 (0.93–0.99)
Proportion of male employees/20% increase		
Between individuals*	0.86 (0.83–0.88)	0.77 (0.74–0.80)
Within individuals**	0.89 (0.87–0.90)	0.79 (0.77–0.80)
Proportion of manual employees/20% increase		
Between individuals*	0.96 (0.94–0.99)	0.92 (0.89–0.96)
Within individuals**	0.93 (0.92–0.95)	0.91 (0.89–0.92)
Proportion of temp employees/20% increase		
Between individuals*	1.01 (0.97–1.06)	1.11 (1.04–1.17)
Within individuals**	1.09 (1.05–1.12)	1.18 (1.14–1.23)
Employee turnover/20% increase		
Between individuals*	1.09 (1.05–1.13)	1.24 (1.18–1.30)
Within individuals**	1.12 (1.10–1.15)	1.24 (1.20–1.28)

* cross-sectional associations in 2000–02; adjusted for age, sex, occupational position, education, residence size, marital status, job contract, self-rated health, and psychological distress.

** longitudinal within-individual associations between 2000–02 and 2004; adjusted for age, self-rated health, psychological distress, and residence size.

Results from the fixed effects models in a subsample analysing a simultaneous change in work unit characteristics and in workplace social capital within individuals in a subsample of 12,108 employees with longitudinal data were consistent with the multilevel analyses. Adjusted for time-varying covariates, individuals experiencing a 20% employee turnover in their work unit during the follow-up had a 24% increased odds of reporting high levels of workplace social capital (OR 1.24, 95% CI 1.20–1.28) and a 12% increased odds of reporting moderate levels of workplace social capital (OR 1.12, 95% CI 1.10–1.15). These associations attenuated but remained significant when all work unit variables were entered in the model (ORs 1.14, 95% CI 1.10–1.19 and OR 1.08, 95% CI 1.05–1.11, respectively). An increase in a proportion of employees with fixed-term contracts during the follow-up was associated with an increase in the level of social capital. However, an increase in size, the proportion of male or manual employees reduced social capital levels. Work unit sickness absence levels did not affect social capital levels.

Results from the ecometric-based analyses at the work unit level were consistent with those from the individual level analyses (Table 3). A 20% increase in employee turnover, compared to no increase, was associated with 1.2-fold increased odds ratio of moderate (OR 1.20, 95% CI 1.09–1.32) to high levels (OR 1.46, 95% CI 1.31–1.63) of social capital in the work unit. A 20% increase in temporary employees in the work unit was also associated with increased likelihood of high levels of social capital in the work unit (OR 1.19, 95% CI 1.06–1.34). Increases in size and in the proportion of male or manual employees were associated with reduced odds of high work-unit social capital. Sickness absence levels at work units were not associated with social capital levels.

**Table 3:** Associations between an increase in work unit characteristics and workplace social capital at the work unit level; odds ratios and their 95% confidence intervals from multinomial regression models in 3090 work units; the Finnish Public Sector Study.

	Moderate vs. low levels of workplace social capital*	High vs. low levels of workplace social capital*
**Work unit characteristic**		
Work unit size/30 person-years' increase	0.96 (0.88–1.04)	0.71 (0.61–0.82)
Percentage of sick leaves/3% increase	0.91 (0.83–1.00)	0.94 (0.84–1.04)
Proportion of male employees/20% increase	0.85 (0.80–0.90)	0.70 (0.64–0.76)
Proportion of manual employees/20% increase	0.96 (0.90–1.01)	0.91 (0.84–0.97)
Proportion of temporary employees/20% increase	1.10 (0.99–1.22)	1.19 (1.06–1.34)
Employee turnover/20% increase	1.20 (1.09–1.32)	1.46 (1.31–1.63)

* cross-sectional associations in 2000–02.

## Discussion

As far as we know, this is the first study to examine whether staff-associated features of the work environment affect levels of social capital at workplace. In the over 3000 Finnish public sector workplaces studied, an increase in the size of the work unit and the proportion of male and manual employees was associated with low, whereas employee turnover (and the proportion of fixed-term employees) was associated with high workplace social capital. The findings were robust to the choice of the level of analysis or method of statistical analysis, and they were not attributable to compositional differences between workplaces. These findings, by suggesting that workplace social capital is contextually patterned, add to understanding of the evolution of social capital in the workplace.

We examined how features of the social environment would influence social capital in the work context. The central proposition of social capital theory is that resources are generated via social interactions within a network of interpersonal relationships and organisational ties; it forms the precondition for the development and maintenance of social capital [6], [34]. Workplaces provide opportunities for sustained interaction, conversations, and sociability, thus creating the foundation for social capital [35]. Our results indicate that an increase in the size of the work unit is potentially harmful to the formation of social capital. The finding parallels those of Ferrie et al. [36] showing that in the public sector moderate expansion in the workforce size was associated with a significantly lower levels social support. Such an expansion in staff number could be due to a merger or restructuring of the work organisation and be followed by changes in the physical environment, for example stemming from a transfer into a new location or increased distance to communal spaces, or due to restructuring of organisational hierarchy. It is hence possible that an increase in work unit size reduces social capital by constraining interaction between co-workers due to changes in the daily social networks, or by inhibiting the maintenance of social capital through social exchange because of new geographical or hierarchical boundaries [35].

Our findings are in part in agreement with previous studies which have investigated how factors of the built residential environment determine social capital. In previous research, urban sprawl, i.e. the spread of a city to outlying low-density areas, has been claimed partly responsible for the decline in social capital in the US [6]. According to the theory, low-density living may create barriers to the formation of neighbourly ties, whereas compact neighborhoods may foster casual social interactions among neighbours [37]. Empirical evidence is still mixed: one study showed that low-density living reduced social capital [14], whereas another reported that urban sprawl may support some types of social capital while negatively impacting the others [15]. Various authors have reported that pedestrian-friendly neighbourhoods encourage local social interaction [16], [17]. However, even this proposition has not received uniform support [18], [19]. Instead, Hanibuchi and colleagues concluded that social capital was determined by the historical age of the community rather than by its walkability in Japan [18]. Indeed, social capital is a multi-faceted concept, exploited in various contexts with multiple determinants, many of which may be context-specific.

In our study, an increase in the proportion of male and manual employees in the work unit were also related to reduced levels of social capital at workplace, irrespective of individual's own sex and SES. Our sample was predominantly female (81%) corresponding to the sex distribution in public sector employees in Finland. Moreover, in half of the workplaces less than 10% of employees were men or manual workers. McPherson et al. [38] suggested that contacts among similar people − with respect to many sociodemographic, behavioural and intrapersonal characteristics − occur more often than contacts among dissimilar people referring to "the principle of homophily". Thus, employees in more homogenous work units may interact more frequently with each other, which in turn generates higher levels of interpersonal trust, reciprocity and social capital [10], [39]. Our findings reflect the fact that like other forms of capital, social capital constitutes a form of accumulated history [35]− here reflecting past investments in social relations which now face an organisational change.

Higher employee turnover and an increase in fixed-term employees were associated with higher levels of workplace social capital, which was an unexpected finding in our study. In general, "birds of passage don't nest" restricting the formation of social ties that take time [6], [8], [40], [41]. Our a priori assumption was therefore that practices like a regular use of temporary employees may undermine the ability of employees to form meaningful relationships at work and hinder the development of social capital [42]. On the other hand, high social capital may enhance mobility and provide better access to information about job opportunities [43]. Thus, it could be that temporary employees are social capitalists, whose social capital developed in another context can possibly be transferred into that of the current workplace [44]. It is also possible that workplace social capital does not necessarily depend on stability. Coleman admitted to this possibility: ”every form of social capital, *with the exception of that deriving from formal organizations with structures based on positions*, depends on stability” [45]. Thus, in a hierarchical work context, social capital may be high even in the face of instability of individuals. Alternatively, the explanation may be that workplaces that employ a high proportion of temporary employees are more strongly focused on performing a specified set of tasks, thereby encouraging strong cohesion in the work unit. This might be the case in teaching hospitals providing internships, for example. At the time of the study, a growing fraction of the workforce in Finland and other OECD countries had contingent or nonstandard jobs, such as part-time or fixed-term contracts [46]. The findings of our study provide some reassurance that this trend is not destroying workplace social capital in the public sector workplaces, although further studies in other contexts (e.g. the manufacturing or service sector) are clearly warranted. Note also that the assessment of social capital at the work unit was mostly based on the responses of permanent employees (over 80% in our sample). Therefore, it is possible that the benefits to the core personnel in long-term jobs derive from good possibilities to use temporary employees when in need, while short-term jobs can create barriers to access to social capital in these work units.

There is currently no universally accepted “gold standard” for assessing social capital at the individual level. However, most studies have assessed aspects of social cohesion, that is individual perceptions of trust and reciprocity as well as reports of civic engagement and social participation as indicators of individual social capital. An alternative approach to measuring social capital is to inquire about network-based resources such as instrumental assistance. For example, the Resource Generator is such an approach; it is made up of an index of different types of instrumental assistance that individuals can access (e.g. getting help with fixing a computer). In our view, the latter approach to measuring “social capital” overlaps a great deal with existing measures of “social support”. Our approach to measuring social capital – in common with most of the studies in public health – is to assess perceptions of social cohesion.

Ecometrics-based measurement of macro-level social capital is in general considered as an improvement in social epidemiology methods [26], [30]. Still, few social capital studies have assessed the ecometric properties of their survey measures of social capital. For example, Mujahid et al [26] studied reported the reliability for their neighbeourhood measures of social cohesion being 0.68 for census tracts and 0.84 for census clusters. The corresponding ICCs were both 0.34. Mohnen et al. [31] found that the average reliability of their neighbourhood social capital measure was 0.62 with an ICC of 3.5%. The work unit social capital measure showed similarly good ecometric properties. The ICC was 0.24 quantifying the percentage of variability in the social capital score that lies between work units. The reliability of the work unit level social capital was 0.71. Similar to ICC, the reliability of the work unit level measure is a function of the between and within-work unit variances, values ranging from 0 to 1. The work unit level reliability relies principally upon the degree of rater agreement and the nubmer of raters per work unit. Therefore it is higher (close to 1) when the work unit means vary substantially across work units or the sample size per work unit is large [26].

### Strengths and Limitations

We note both strengths and limitations to our study. With over 43,000 participants, the sample size was large, and we were able to accurately link them to their work units to measure contextual features of workplaces objectively, thus, avoiding information bias. Using ecometrics we could tease out bias due to measurement error when the perception of social capital is influenced by the characteristics of the individual respondent. However, we do not know the extent to which the modelled increase in the exposure variables reflected real-life changes (rather, they reflect pre-existing variations across work units). Nonetheless, in fixed effects analysis the majority of participants experienced changes of comparable magnitude. Indeed, repeated measurements within individual over two points in time are a strength of the study. Loss of participants due to missing values in any of the variables measured was relatively modest (0–3%) and is an unlikely source of bias in our results. Most social capital studies do not simultaneuously assess social capital as an individual attribute and as a property of the collective but rather tend to assess one or another depending on their method of measurement. Therefore, a further strength of this study is that we assessed social capital both at the individual level and the work unit level.

Although we controlled for an array of individual factors, (including individual level counterparts of the contextual variables) the possibility of bias due to unmeasured factors cannot be ruled out. Our analysis was limited to administrative work units and we did not attempt to delve beyond these official boundaries into informal groupings at work. Personal attitudes, relative to work unit factors, may also play a role in predicting social networks in the workplace. In the present study, our measure of social capital combine vertical aspects, such as trust in the supervisor, and horizontal aspects, such as social contacts, cooperation and trust between colleaques in the workplace) [47]. In future studies, it is important to examine the extent to which these two aspects of social capital are determined by a distinct set of contextual factors. The participants were working in Finnish public sector workplaces, and the majority of them were women which may compromise the generalizability of the results to male-dominated populations and other branches of industry.

### Conclusions

In this paper, we have sought to highlight workplace characteristics that potentially influence levels of social capital. The relevance of these findings lies on the fact that many of the changes we modelled are on-going in the workplaces. The findings may direct us to possible intervention to boost participatory and cooperative behaviour in the workplace. Such workplaces have space for conversation, action and interaction; occasions for the more-or-less planned coming together of people and their ideas to build or extend networks of connections and therefore, the stocks of social capital [38].
